# Toward Safe and Ethical Implementation of Health Care Artificial Intelligence: Insights From an Academic Medical Center

**DOI:** 10.1016/j.mcpdig.2024.100189

**Published:** 2024-12-20

**Authors:** Austin M. Stroud, Michele D. Anzabi, Journey L. Wise, Barbara A. Barry, Momin M. Malik, Michelle L. McGowan, Richard R. Sharp

**Affiliations:** a Biomedical Ethics Research Program, Mayo Clinic, Rochester, MN; b Robert D. and Patricia E. Kern Center for the Science of Health Care Delivery, Mayo Clinic, Rochester, MN; c Center for Digital Health, Mayo Clinic, Rochester, MN

## Abstract

Claims abound that advances in artificial intelligence (AI) will permeate virtually every aspect of medicine and transform clinical practice. Simultaneously, concerns about the safety and equity of health care AI have prompted ethical and regulatory scrutiny from multiple oversight bodies. Positioned at the intersection of these perspectives, academic medical centers (AMCs) are charged with navigating the safe and responsible implementation of health care AI. Decisions about the use of AI at AMCs are complicated by uncertainties regarding the risks posed by these technologies and a lack of consensus on best practices for managing these risks. In this article, we highlight several potential harms that may arise in the adoption of health care AI, with a focus on risks to patients, clinicians, and medical practice. In addition, we describe several strategies that AMCs might adopt now to address concerns about the safety and ethical uses of health care AI. Our analysis aims to support AMCs as they seek to balance AI innovation with proactive oversight.

Motivated by deep learning and electronic health record advancements in the early 2000s,^1^ the prospect of supporting clinical decision-making and reducing administrative burden in various specialties has contributed to an unprecedented wave of popularity for artificial intelligence (AI) in health care.^2^, ^3^, ^4^ Despite its much-lauded potential, there are considerable challenges with translating AI models from research to the bedside.^5^ Issues, such as validation, inequity, and explainability have undercut the potential for many systems to live up to their hype.^6^^,^^7^ These issues along with other potential risks have placed AI (in health care and beyond) on a collision course with greater public scrutiny and regulation because of growing safety and equity concerns.^8^^,^^9^

Public alarm over AI has mounted because of the potential risks these technologies pose to society, ranging from more fantastical and ideological claims of artificial superintelligence^10^ to more relevant considerations of enabling coercive and exploitative labor practices.^11^^,^^12^ However, in responding to public unease, there are certain risks that warrant prioritization in a health care context. Three key concerns, driven by specific incidents, are around poor performance,^13^ racial bias in resource allocation decisions,^14^ and uncertainties in data handling.^15^^,^^16^ These justifiably lead to skepticism over further integrating AI systems into health care, particularly systems that can inform or drive clinical decision-making or care management (eg, clinical/patient care decision support systems). In addition, many of these are black-box systems—in which the rationale behind model outputs is unintelligible to an end-user—raise concerns about the use of these technologies in clinical decision-making.^17^^,^^18^ To address these concerns, governing bodies such as the United States Food and Drug Administration (FDA) are signaling changes to the regulatory landscape^19^ with implications for academic medical centers (AMCs) and other organizations at the forefront of developing AI systems.

Currently, there is a lack of consensus on health care AI ethics and safety standards in what is an underdeveloped regulatory landscape. For instance, the FDA’s plan for software as a medical device^20^ may apply to some AI systems classified as medical devices but not others, such as those involving novel uses of large language models.^21^^,^^22^ As hubs for innovation, AMCs must navigate considerable development and implementation challenges in weighing potential benefits and harms of integrating AI into health care delivery.^23^^,^^24^ This unique positioning may also be a proving ground for developing meaningful ethical and regulatory standards for the implementation of health care AI.

In this article, we highlight several potential harms that accompany health care AI. We describe the difficulties of deploying AI ethics principles in health care settings and present a set of actions that AMCs may take to advance a culture of ethical and safe health care AI—meaning that all appropriate measures have been taken to reduce harm, ensure effectiveness, and promote equity. If there is a culture in which technological developers and AMCs prioritize rapid development of health care AI without adequate safeguards, the concerns outlined earlier will only be exacerbated. Recognizing that the integration of AI into health care is in relatively nascent stages, we suggest that the steady and iterative implementation of AI ethics through practical approaches will be critical to developing a culture of AI ethics and safety at AMCs.

### Contextualizing Potential Harms

To implement AI safely and ethically, it is critical that health care institutions anticipate and mitigate potential harms to patients, clinicians, and medical practice (Table).

**Table:** A Categorization of Harms AI poses in Several Domains of Health Care.

Domain	Potential Harms
Patients	Model inaccuracies leading to adverse outcomes
	Limitations to autonomy
	Population health issues pertaining to equitable and fair health care
Clinicians	Personal accountability for following or disregarding AI recommendations
	Loss of clinical expertise due to overreliance on AI technologies
	Additional burden incorporated into clinician workflows
Medical practice	Losing the human element of medicine in clinician-patient interactions
	Failure to fulfill expectations of safe and efficient care
	Inability to meet institutional standards
	Tacit acceptance of exploitative commercial labor practices
	Contributing to environmental impacts and related health disparities

Abbreviations AI, artificial intelligence.

### Harms to Patients

A prominent concern is the potential for AI to introduce harm to patients’ health and wellbeing. Artificial Intelligence models similar to all models (and, indeed, human decision-making), are never perfectly accurate. However, there is an issue when they are less accurate than expected, which can result from unrepresentative or biased training datasets.^25^^,^^26^ The widespread implementation of AI systems may also have adverse consequences for certain patient communities and populations. Limited sociodemographic representation in model training data can exacerbate bias and negatively impact patient populations who are underpresented or misrepresented in a model’s design.^27^ This can also concentrate clinical benefits to overrepresented populations while failing to serve underrepresented patient groups.^28^ Of particular concern is the potential to create “AI exclusion cycles”—when discrimination by clinicians (undergirded by big data and AI systems) leads minoritized patients to develop strategies that clinicians misconstrue, thereby reinforcing discrimination.^29^ These problems are not necessarily unique to AI, but the data-mining nature of AI models, in contrast to previous substantive statistical modeling, can obscure the need to reason about such latent cofounders and underlying constructs vs measures. Downstream consequences of embedded bias could include compounding injustice,^30^ which may damage relationships between health systems and affected patient populations.

There is also the risk that the introduction of AI systems may magnify the digital divide—disparities in access to digital technologies and infrastructure—for underserved patient populations by adding more complex technologies atop underlying systemic inequalities in access^31^ There may be an unfair distribution of benefits and harms across patient subpopulations as AI integration could result in greater digital exclusion of groups who already face inequitable access to digital technologies.^32^ As AMCs consider broader implementation of AI, assessments of whether these endeavors are beneficial to the patients and communities they serve will be paramount. Moreover, communities whose perspectives have historically been marginalized in health care should be included when making these assessments. Ideally, these inputs should be treated as central to the technological design of health care AI as opposed to being mere afterthoughts.

Beyond direct harms to patients’ health and wellbeing, there are potential dignitary harms—nonphysical injuries involving disrespect to persons, including constraints on patient autonomy—that may accompany health care AI.^33^ Limitations to patient autonomy may occur when medical AI systems do not account for pluralistic value alignment.^34^ An example is how multivariate models for resource allocation may, during the decision-making process, choose a certain value of trade-offs between quantities such as time efficiencies, cost management, or disease-free survival that may be contrary to the patient’s values and wishes.^34^ Moreover, there also may be unanticipated consequences in the art and practice of medicine that patients find unfavorable, such as a loss of autonomy should these technologies be integrated into their care without their consent, as in the surreptitious use of an AI model to analyze a patient’s electronic health record data.

### Harms to Clinicians

As primary end-users of AI systems, clinicians are exposed to potential risks resulting from the output of clinically actionable information. Model errors or inappropriate use of model outputs could raise personal responsibility and legal liability concerns for clinicians leveraging AI in clinical decision-making.^35^ Others have raised concerns regarding the potential for physicians to depend too heavily on AI systems, resulting in de-skilling and automation bias^36^ that can be harmful to their overall practice. Ultimately, physicians are accountable for the welfare of their patients, which may be threatened if physicians put too much trust in technology that they do not fully understand when crafting patient care plans.

Despite optimistic claims that AI will reduce the administrative burden faced by physicians, in practice, they may increase this administrative burden.^37^ Moreover, AI systems may introduce new obligations for physicians. For example, patients expect physicians to be able to explain and address safety concerns for how medical AI technology works,^38^ which may not be possible with black-box medicine.^39^ This limits physicians’ ability to clinically reason about appropriate uses of these AI systems and may lead to erroneous judgments that harm patient welfare. Ultimately, patients entrust physicians to act in their best interests, and if physicians’ utilization of medical AI tools fails to do so when caring for them, this can violate this trust and harm the physician-patient relationship.^40^

### Harms to Medical Practice

Beyond harms to patients and clinicians, there are potential harms to the practice of medicine. Although these harms may be more abstract than direct risks to individuals, their potential impacts remain relevant and warrant ethical consideration. For instance, patients want to know that their physician cares for them and empathetically hears their concerns.^40^ However, although greater automation of health care interactions might improve efficiencies in health care delivery, it may erode the human element of medicine that both patients and clinicians consider essential and is relevant to clinical outcomes,^40^^,^^41^ such as the redesignation of tasks traditionally undertaken by clinicians to a health care AI system and decision-making processes based on patient concerns. This deviation from current practice may disrupt the clinician-patient relationship in unforeseen ways as AI becomes more common in clinical interactions.

An additional consideration is when erosion of trust extends to the reputation of the health care system. AMCs need to be proactive in establishing transparency about values and priorities when designing and implementing AI technologies. One example is data stewardship. The acquisition and sharing of patient data by AMCs to develop digital health technologies, particularly without patients’ awareness,^16^ represents a practice in AI model development that could be considered a violation of the fiduciary relationship between patients and health care organizations (without appropriate disclosure and patient authorization). Such a violation could reinforce patient concerns that a health care system is not fulfilling its obligations to provide appropriate patient-centered care, especially if these systems are commercialized. Cases of insurance companies denying medical claims based on algorithmic recommendations raise similar concerns regarding the potential adoption of these clinical decision support systems to support profit motives at the expense of quality health care.^42^ This would be particularly worrisome to patients who harbor concerns regarding their health care costs and insurance coverage.^38^

Finally, there is consideration for diffuse harms involved in operationalizing AI in medical practice that undermine the aims of health care. With much of the current technological infrastructure for health care AI being reliant on commercial partnerships,^43^ there is a tacit reliance on industry practices. This includes employing data workers from low-income and middle-income countries who are often underpaid and exposed to distressing content without adequate mental health resourcing.^12^^,^^44^ Furthermore, the training and deployment of AI models demands intense energy consumption that contributes to environmental harms.^12^^,^^45^ These environmental harms have been linked to racial and health disparities exacerbating health risks for many historically marginalized patient populations.^46^ Although these impacts may be invisible to health care institutions that deploy AI systems, it is worth interrogating how assessments of the potential benefits of AI to medical practice are weighed against these and other indirect harms to populations that may already be experiencing disproportionate health risks. AMCs may also have strategic interests in ascertaining these environmental harms given recent initiatives targeting sustainability and climate action^47^ and addressing longstanding ethical concerns regarding health care waste.^48^^,^^49^

### Responding to Safety Concerns

A lack of clear and cohesive regulatory standards that keep pace with technological advancements is a notable gap for health care AI.^50^^,^^51^ Some current regulations, such as Section 1557 of the Affordable Care Act, offer concrete considerations regarding nondiscrimination in the use of patient care decision support systems by health care organizations.^52^ Guidance by the FDA regarding AI in software as a medical device also offers risk-based market regulation.^20^ However, even these hard law approaches, although clearly integral to safety and ethics, possess limitations in their specificity^53^ and scope.^54^ Regulatory authorities in the United States have recognized this gap and have signaled action with various reports^19^^,^^55^ and executive actions.^56^^,^^57^ Communication from the FDA has even outlined a total product lifecycle approach to health care AI oversight involving the adaptation and expansion of current regulatory authority and a critical role for stakeholders like AMCs in evaluating AI system performance.^58^

Although these and other regulatory developments will undoubtedly play a significant role in shaping future best practices for the safe implementation of health care AI, the current implications of these position statements will remain unclear until a cohesive oversight plan is enacted and enforced. As regulators grapple to define the legal and regulatory mechanisms that will inform AI implementation, AMCs and other health care institutions are left to determine what strategies and structures best meet their current needs and those of the patient populations they serve.^24^

Deciding what to do; however, is a rather complex task. There is a deluge of scholarship attempting to situate ethical principles and guidance, with virtually every group, from multilateral organizations^59^ to technological firms^60^ to professional societies^61^ opining on their vision for AI ethics. The breadth of this scholarship is notable for contributing varying perspectives on how issues with AI systems should be assessed and mitigated. One example includes the application of research ethics frameworks suggesting a multistage approach that adheres to clinical trial design and evaluation principles.^62^ However, a differing view highlights the limitations of such research and legal frameworks with regard to justice, equity, and oversight.^63^ This scholarly literature highlights several competing principles whose interpretation will be critically important to finding consensus on guiding ethical frameworks, priority issues, and governance strategies.

Despite a myriad of perspectives on the ethical implementation of AI, several reviews have identified emerging points of consensus regarding key ethical principles for AI implementation, with transparency, for example, being frequently highlighted.^64^^,^^65^ In relation to existing bioethics scholarship, Floridi et al^66^ have charted similarities between principles described in many AI ethics frameworks and those in medical ethics—such as the principles of beneficence, nonmaleficence, autonomy, and justice. However, other work reviewing empirical studies has noted misalignments and knowledge gaps between principle-informed frameworks and stakeholder perspectives.^67^ It is difficult to identify an emerging consensus on specific actions that should be taken now, as these principles may be interpreted differently across stakeholders and circumstances.^64^^,^^68^

Some scholars have challenged the value of proposed AI ethics principles, characterizing those principles as either meaningless, isolated, or toothless with limited feasibility for implementation.^69^ Similarly, Mittlestadt contends that the application of ethics principles to health care AI is fraught with practical challenges given the stark differences in technology and medical applications.^65^ Others have noted the potential of ethics washing—espousing principles for the sake of acknowledgment but not supporting them with concrete action—presenting the illusion of responsible governance practices.^69^^,^^70^ This discontent with the practical value of AI ethics highlights a clear need for implementation strategies that define specific actions that can be taken in support of safe, and ethical deployment of health care AI.^71^

Taking these proposed ethical frameworks and critical perspectives into account, in what follows we describe several specific actions initiated at one AMC in response to growing public concerns about the safety of health care AI. We view these strategies as consistent with a range of ethical principles and guidance. These approaches aim to cultivate an informed, action-oriented context in which concerns about AI ethics can be discussed transparently with input from multiple stakeholders. It is important to note that we do not offer these suggestions as best practices or consensus strategies but rather as one AMC’s efforts to develop AI governance strategies in the absence of clear regulatory policies and guidelines.

### Strategies for the Ethical Adoption of Health Care AI

In what follows, we describe several strategies employed at one AMC that might be adopted in support of the ethical deployment of health care AI. These include: (1) supporting translational ethics research, (2) creating specialized advisory and governance structures, and (3) expanding education on AI ethics and safety. Although each individual strategy may be limited in its scope and impact, the collection of these initiatives supports a multifaceted approach to AI adoption. The approaches we describe are not traditional governance structures but aim to promote an organizational culture that promotes AI ethics and safety across all stages of research, development, and deployment (Figure).^72^

**Figure:**
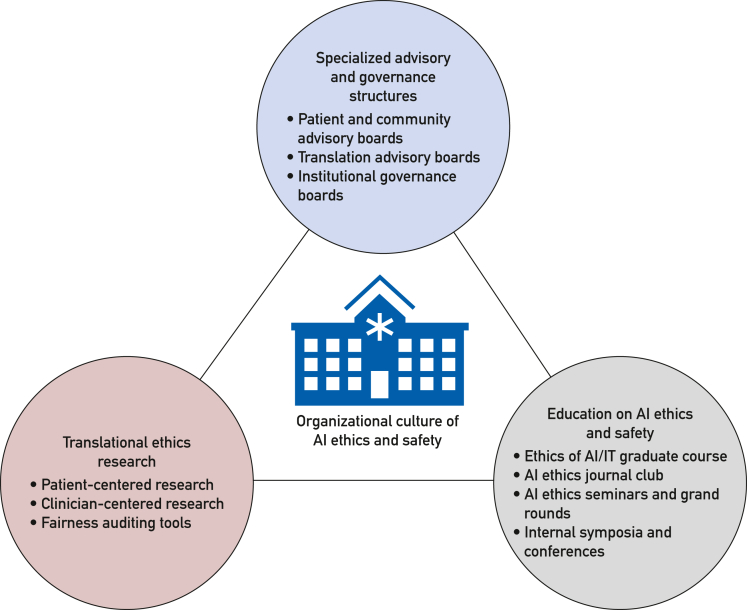
A mapping of approaches to build a culture of AI ethics and safety at an AMC.

### Supporting Translational Ethics Research

Translational ethics research seeks to evaluate moral perspectives on emerging technologies and presents one approach for assessing stakeholder values regarding healthcare AI.^73^ Such research has been noted as a proactive method for encouraging the design and implementation of these technologies.^74^ At Mayo Clinic we have previously conducted patient-centered qualitative research characterizing patient concerns about AI in their health care including apprehensions around safety, costs, and data stewardship.^38^ This research has also provided a conceptual model for understanding patients attitudes toward AI systems within the broader goals of health care.^75^ We have also performed research examining patient preferences for information about AI systems, which has informed initiatives on trust and transparency.^76^ Other physician-centered research has reported specialist perspectives on AI technologies,^77^^,^^78^ characterizing physician receptivity toward the adoption of AI systems. Studies such as these applying social science and bioethics research methods can support the direction of model development and institutional decision-making with consideration for stakeholder concerns.^79^ Furthermore, stakeholder perspectives are relevant to regulatory guidance, as they have been previously considered in policy discussions by the FDA regarding appropriate transparency for health care AI systems.^80^

In addition to stakeholder-focused research, there is a role for translational ethics research that seeks to develop tools for the practical application of ethical principles. These implementation-focused tools can play a role in how AMCs can address criticisms of AI ethics feasibility and is another key focus at Mayo Clinic.^71^^,^^81^ Currently, there is a spate of technical tools that focus specifically on auditing model performance to see if a model performs worse on certain demographics or patient characteristics vs its performance on a majority group by the same metric. The effective use of these tools requires a choice of a quantitative operationalization of fairness, and all such operationalizations are deeply limited,^82^^,^^83^ and should be understood as one component of ethical considerations. The absence of quantitatively measured bias is insufficient for a system to be acceptable, but it is necessary, as such auditing can provide concrete evidence of the existence and magnitude of disparate impacts.

Although translational ethics research serves an important function in AI implementation, funding for such research has been limited when compared with direct technological investments.^84^ As AMCs and other health systems consider investments in AI projects, they should similarly consider appropriate resourcing and funding for addressing ethics and safety concerns. Although translational ethics research does not guarantee the safe and ethical implementation of AI, we note this strategy as one avenue for prospectively identifying harms that may impact relevant stakeholders. As institutions leading health care AI development whether internally or by partnerships,^23^ AMCs are well situated and resourced to advance this research. Translational ethics research can reveal unspoken ethical concerns raised by novel technologies and support the formation of policy responses.^85^ Furthermore, additional work in this area serves as an opportunity to bridge the disconnect in high-level principles and empirical scholarship and connect multiple stakeholders necessary to inform AI safety and ethics.^67^ By supporting translational ethics research, institutions can foster the development of pragmatic tools and practices and inform decision-making on AI adoption.

### Creating Specialized Advisory and Governance Structures

Another step undertaken at our AMC is the establishment of advisory boards for healthcare AI initiatives.^86^^,^^87^ These formal bodies aim to support the deployment of AI systems by embedding stakeholder evaluations in research, development, and implementation.

Patient and community advisory boards are one example. Banerjee et al^88^ articulate the advantages of this approach, noting that co-designing AI systems with patients is critical to fostering trust and managing expectations among involved stakeholders. At Mayo Clinic we have established and maintained such a patient and community advisory board—the Health Data and Technology Advisory Board—that advises institutional leadership on health care AI and digital health technologies.^89^ Board members include patients and community members with a variety of health care and technological experience. Researchers, clinicians, or health care administrators can present specific initiatives to the board, whose members will then provide their perceptions and concerns. The board has also developed an evaluation framework consisting of key areas of interest when assessing AI projects from patient and community perspectives.

A related group established at Mayo Clinic is the enterprise AI translation advisory board, which assesses whether an AI technology is suitable for clinical use and assesses requirements to achieve successful translation.^90^ Advisory board members include multidisciplinary subject matter experts from areas such as medicine, data science, biomedical ethics, and implementation science. Internal advisory boards such as this can assist in assessing the impact of AI technologies on patient care activities and clinical workflows. Board members conduct contextual research, which can reveal the degree of buy-in from the intended users, or where a proposed system targets an irrelevant or inappropriate part of a clinical workflow. In addition, board members evaluate the technical performance and appropriateness of AI algorithms and examine their impact in real-world settings,^91^ providing a mechanism for ensuring their safe deployment.

In addition, Mayo Clinic has established a higher-level governance structure charged with evaluating institutional risks associated with health care AI known as the Biomedical Ethics and AI Advisory Council. The council is charged with advising AMC leadership and creating institutional policies. In addition, this board assesses the potential reputational risks involved in the adoption of large AI-enabled systems in clinical practice and their alignment with patient needs and organizational values. The board also reviews published external AI ethics guidance documents and identifies their relevance for the institution. By situating AI governance within broader organizational context, the council is well positioned to advise on internal policies, standards, and practices that help to promote an organizational culture of ethics and safety. Members of the council also consult on ethical issues raised by AI systems that emerge during research and patient care.

### Expanding Education on AI Ethics and Safety

AI ethics education fits within a broader context of growing demands for educational resources as AI becomes further deployed in health care settings.^92^, ^93^, ^94^ At Mayo Clinic we have developed and facilitated a graduate course for scientists and health care professionals titled “Ethical Issues in Artificial Intelligence and Information Technologies,” intended as a formal educational resource for approaching AI ethics. The course has been offered annually since 2021 and covers historical context and current topics related to the ethical, legal, and social implications of AI in medicine across the translational research continuum. Learners who have participated in the course include medical students, graduate students, residents, fellows, attending physicians, pharmacists, basic scientists, and nurses. The case-based curriculum equips learners with the skills to identify and propose tactics to address ethical issues in AI and information technology as they arise in clinical practice and research. In addition, the authors have piloted and maintained an institution wide AI ethics journal club to facilitate conversations with multidisciplinary AI stakeholders. The journal club has addressed key topics such as the black-box problem,^17^ the utility of AI ethics,^69^ and impacts of AI on patient-centered care.^34^

We have also taught on topics related to health care AI ethics in an internal education seminar series and in various grand rounds lectures. These presentations reach audiences of clinicians, investigators, trainees, allied health professionals, and students, and topics have included normative questions surrounding the role of ethics; limitations of noncausal outcome-only modeling; and a primer on ethical issues in health care AI and information technologies.

We have also presented at several internal, institution wide conferences and symposia focused on AI, with the intention of bringing AI ethics content to typically technically focused spaces. These venues have provided the opportunity to expand the reach of AI ethics content and network with institutional stakeholders who may not find themselves in traditional classroom settings. By creating and promoting various forms of ethics-focused content, we hope to complement more technically focused educational resources that are already available at the institution. Future educational endeavors might focus on broadening the scope of activities to support patient education materials, public dialog and accessible content that may increase public familiarity with advances in AI and its applications in health care.

### Limitations and Future Directions

As noted above, we do not wish to suggest that these strategies should be universally adopted or represent best practices for AI implementation. In addition, the analysis and approaches we present are limited in several ways. First, our discussion of harms, ethics, and approaches is nonexhaustive, as there may be nuanced considerations for specific AI models and specialty contexts. Second, the practical applications we report pertaining to empirical research and institutional activities are contextual to a single multisite academic health system and may not be generalizable to other health systems. Moreover, aspirational goals such as building a culture of AI ethics and safety will require substantially more effort than the initial steps mentioned above and will vary by organization. The approaches we report may also evolve and adapt over time to meet the changing institutional needs. There are differing and potentially more effective ways of approaching safe and responsible AI from various situational and cultural contexts better suited for diverse end-users and patient populations.^95^ Finally, our perspectives are framed by a professional stake in enabling ethical implementation of AI at an AMC in the United States, whether through the promotion of AI technologies or advocating against inappropriate applications.

As leaders of AMCs consider the safe and ethical implementation of health care AI, we note that our approaches may not be feasible in other settings due to a range of circumstances. Although institutions can focus on immediate and broad policy responses, we hope that our approaches illuminate potentially nuanced ways to address AI safety and ethics through an adaptive institutional culture. For instance, staff education initiatives can be iteratively refined to support ongoing change management efforts. We also suggest that AMCs and other health institutions report on their own approaches for safe and ethical health care AI (regardless of whether they are successes or failures). Sharing innovative strategies and considering opportunities to pool resources across institutions may support long-term implementation and patient safety efforts.

## Conclusion

As AI gains a stronger presence in biomedical research and the practice of medicine, health care institutions will have to ascertain the relative impacts to patient and professional populations. We approach the issue of AI ethics and safety by considering several potential dimensions of harm and report attempts at building a culture of AI ethics and safety at an AMC. Although no single ethical framework, guidance document, or implementation strategy will alleviate the risks accompanying health care AI, bridging the gap between principles and practice will help support the implementation of safe and responsible AI. To this end, we present several options for implementing AI ethics that can be piloted and reported by health systems to further develop an understanding of potential approaches toward responsible health care AI adoption.

## Potential Competing Interest

Dr. Barry is funded in part by the US Food and Drug Administration and Anumana, Inc. All other authors have no conflicts to disclose.
